# Kidney transplantation versus maintenance dialysis in 14 EU countries: cost savings, payback time, and budget impact

**DOI:** 10.3389/fpubh.2026.1801439

**Published:** 2026-03-10

**Authors:** Horia Iuga, Mehmet Ali Balcı, Simion Mihon, Ömer Akgüller, Florin Ioan Elec

**Affiliations:** 1 Faculty of Medicine, Iuliu Hațieganu University of Medicine and Pharmacy, Cluj-Napoca, Romania; 2 Department of Mathematics, Faculty of Science, Mugla Sitki Kocman University, Mugla, Türkiye; 3 Department of Finance-Accounting, “1 Decembrie 1918” University of Alba Iulia, Alba Iulia, Romania; 4 Department of Urology, Iuliu Hațieganu University of Medicine and Pharmacy, Cluj-Napoca, Romania; 5 Clinical Institute of Urology and Renal Transplantation, Cluj-Napoca, Romania

**Keywords:** cost-effectiveness analysis, dialysis, health economics, kidney transplantation, living donor transplantation, neural ordinary differential equations

## Abstract

**Purpose:**

To assess the economic benefit and payback period of kidney transplantation compared to maintenance dialysis across 14 European Union countries (2019 and 2023), with a focus on living-donor transplantation.

**Methods:**

A longitudinal country panel was compiled from European registry and national report. Cost–benefit analysis calculated annual net savings and break-even time, and cost-utility analysis estimated quality-adjusted life year gains using published utility weights with survival adjustment. A Graph-Regularized Neural Ordinary Differential Equations framework was introduced to capture nonlinear temporal dynamics and cross-country interactions, and its predictive performance was benchmarked compared against linear cost–benefit calculations and two-way fixed-effects regression using root mean squared error.

**Results:**

Transplantation was economically dominant in all country-years. Mean annual net savings were $37,471 and mean break-even time was 3.12 years (0.5–6.8). Incremental quality-adjusted life years averaged 4.38. Countries with living-donor share above 20% had shorter payback than those at or below 20% (2.01 vs. 4.28 years). The findings support the widespread economic superiority of transplantation over dialysis across the healthcare systems of all nations under investigation.

**Conclusion:**

Living kidney transplantation consistently offers cost savings and health benefits across all health systems in the European Union. However, its scalability depends on country-specific payback periods, which are primarily influenced by upfront transplant costs.

## 1 Introduction

Chronic kidney disease (CKD), which affects an increasing number of people and has severe clinical and financial consequences for healthcare systems, is one of Europe’s most pressing public health issues. When chronic kidney disease (CKD) advances to end-stage kidney disease (ESKD), kidney replacement treatment (KRT) is required, either in the form of maintenance dialysis or kidney transplantation. These therapies impose significant and continuous costs on publicly supported healthcare systems (1, 2). The number of individuals requiring long-term renal replacement therapy has steadily increased across European nations due to factors such as aging populations, higher rates of diabetes and hypertension, and improved survival among patients with chronic diseases (3, 4).

KRT is a perfect example of how cost structure is just as important from a financial standpoint as clinical efficacy. While transplantation usually concentrates costs in the peri-transplant period, followed by relatively lower (but ongoing) post-transplant care and immunosuppression costs, dialysis entails persistent, recurrent costs that scale directly with prevalence (5, 6). Because it allows a direct comparison of the financial burden associated with different KRT procedures under a similar accounting logic, Cost–Benefit Analysis (CBA) per patient becomes an important policy framework. When scaled to national prevalence levels, the yearly per-patient cost savings following transplantation (compared to dialysis) provides an actionable indication for health budget planning.

Given that KRT programs can be among the most costly aspects of managing chronic diseases in publicly financed systems, the economic implications of modality selection have taken center stage in health policy discussions. Dialysis-related spending is a significant sustainability concern, according to recent European policy-oriented evaluations (7). This highlights the need to find solutions that safeguard both clinical results and financial viability. Current data also show significant heterogeneity and evolving trends in transplantation costs globally, indicating that cost dynamics should be closely monitored and interpreted in light of financing constraints and health-system capacity (8).

In light of this, payers and health ministries might use per-patient economic evaluation as a useful tool for decision-making. Clinical paths may be translated into similar financial metrics through a patient-level Cost–Benefit Analysis. These metrics include (i) the total yearly treatment costs per patient under dialysis vs. transplantation and (ii) the annual cost savings per patient following transplant (compared to dialysis). Crucially, decision-makers also need a clear estimate of the break-even time (the number of years needed to recoup the original transplant cost through future yearly savings) because transplantation involves substantial initial operation and peri-operative expense. This “payback horizon” facilitates longer-term planning and investments in transplant infrastructure and staff while also influencing whether transplantation scale-up is deemed financially feasible during short-term budget cycles (9, 10).

However, without a clear evaluation of how modifications to the treatment mix affect overall national spending, per-patient savings do not automatically transfer into system-level affordability. Therefore, by assessing the financial effects of putting policy scenarios (like boosting transplant activity) into practice within practical restrictions, Budget Impact Analysis (BIA) enhances CBA.

In this context, the present study examines the cost–benefit analysis of kidney transplantation compared to maintenance dialysis in European countries over the period 2019–2023, using a harmonized framework that integrates comparisons of per-patient costs and perspectives of system-level budgetary impact. To achieve the proposed objective, the study addresses the two main research questions: *1. What are the annual per-patient net cost savings of kidney transplantation versus maintenance dialysis? 2. What is the break-even time (payback period) required for cumulative post-transplant savings to offset the upfront transplantation cost, and how does it* var*y across countries (2019 vs 2023)?*

Europe offers a useful context since the continent benefits from registries and uniform reporting, and health systems there are generally similar in their dependence on public funding. Because only these nations provided comprehensive and comparable data for the essential variables needed for the economic evaluation (modality-specific costs, patient volumes, and budget indicators) during a shared window (2019–2023), we limit the study to 14 EU member states. Because member states typically report health expenditure and budget indicators within harmonized methodological frameworks and because the EU institutional environment increasingly encourages convergence in health technology assessment, concentrating on the EU also strengthens policy relevance.

In this study, we exclusively focused on Living Donor (LD) transplant data, intentionally excluding Deceased Donor (DD) figures from the primary cost-effectiveness model. This distinction was made to minimize the heterogeneity inherent in transplant logistics; while DD rates are heavily influenced by external variables such as mortality rates, intensive care unit capacity, and opt-out legislation, LD transplantation represents a planned, elective intervention. By isolating LD data, we aim to more accurately evaluate the direct impact of healthcare policy and resource allocation on transplant activity, independent of the stochastic nature of cadaveric organ supply.

This study offers an original contribution in several significant ways. Firstly, it delivers a harmonized, cross-country economic evaluation of living-donor kidney transplantation versus maintenance dialysis across 14 European countries (2019–2023), generating policy-actionable per-patient indicators (annual net savings, break-even time, and discounted net present value) while also incorporating quality-adjusted outcomes through a cost-utility (QALY/ICER) framework. Secondly, it introduces and validates a Graph-Regularized Neural ODE approach tailored to sparse two-time-point health-economic panels, leveraging cross-country similarity constraints to capture nonlinear dynamics and improve predictive performance beyond conventional linear CBA and fixed-effects benchmarks. Thirdly, it translates these micro-level parameters into system-level decision support by combining dynamic regime/trajectory diagnostics (cluster transitions and financing “lock-in” patterns) with structured budget-impact scenarios (+10%, +25%, +50%) that quantify the fiscal implications of scaling transplantation within publicly financed health systems.

## 2 Literature review

Whether kidney transplantation is a more economical alternative for renal replacement treatment than maintenance dialysis is the primary clinical decision that healthcare authorities must make. Even while clinical evidence (11–13) regularly demonstrates improved survival and quality of life following transplantation, the economic case remains complex because to the substantial upfront investment requirements that must be weighed against long-term operational savings from avoided dialysis expenditures.

The first year after a transplant is often more costly than ongoing dialysis, but expenses drop significantly in subsequent years, leading to net savings over a relatively short period, according to recent European empirical research. Zhang et al. (14) used Swedish longitudinal register data and causal-inference methods to find higher costs in the first-year post-transplant compared with dialysis, followed by notable cost savings in years 2 and 3. In cases with high dialysis expenses, this pattern is common and suggests a relatively quick return on investment (14). A study by Agüero-Cobo et al. (9) revealed significant regional differences in transplantation costs across Spain’s health systems, indicating that “one national estimate” may obscure important variations relevant to policy even within the same country. Furthermore, a benchmark analysis from Sicily (Italy) linked transplant performance to financial outcomes and underscored the importance of local organisation and throughput for actual savings, showing how increasing transplant activity could lead to measurable health system benefits (15). However, because economic evaluations often differ in perspective (payer versus societal), cost components (such as procurement, medications, and donor expenses), time horizon, and approaches to managing selection bias in transplantation, comparability between studies remains limited. A recent systematic review emphasising the need for standardised, transparent methods when comparing nations examined methodological diversity in kidney-transplant economic evaluations and found substantial variation in quality assessment processes (16).

In Europe, there are notable and persistent differences in transplantation rates across countries, caused by both institutional or demand-side factors (referral patterns, listing policies, and financial incentives) and supply-side limitations (donor availability, infrastructure, and staffing). Most nations still perform fewer living-donor transplants than deceased-donor transplants, and there is considerable variation in overall transplant rates, as shown by ERA-registry data highlighting unequal progress and a distinct East–West gradient (17). Financing arrangements may unintentionally encourage dialysis expansion from a health-economic point of view. Cross-country data supporting induced demand in kidney replacement therapy (KRT) is presented by Redeker et al. (18). This indicates that provider incentives and capacity might influence treatment volumes beyond clinical needs, which directly impacts efforts to increase transplant numbers.

According to a scoping review of dialysis payment systems, reimbursement models (such as fee-for-service vs. bundled payments) have an impact on provider behavior, modality mix, and possibly cost trajectories. This supports the claim that modality policy and KRT funding are inextricably linked (19). Legislative frameworks for dead donation (opt-in vs. opt-out) have generated much discussion on the supply side. However, mixed-methods data shows that default law by itself has inconsistent results; any increases in donation and transplantation are probably mediated by public trust, family-approach practices, and implementation quality (20). When considered together, the European policy literature increasingly suggests a “package” perspective: whether transplantation can replace dialysis at scale depends on organizational capacity, incentives, and governance (17, 19).

Because it reduces waiting times, makes planned surgery easier, and allows for preemptive transplantation before dialysis begins, living-donor kidney transplantation (LDKT) is both strategically and clinically valuable. This reduces cumulative dialysis exposure and associated morbidity and expenses. LDKT outperformed deceased-donor paths in a sizable European registry cohort of older adults receivers, highlighting its high-value function when practical (21). Preemptive living donation is presented as an effective population-health method with strong protections (22).

Policy instruments that expand living donation (such as paired exchange, compatible-pair programmes, donor protection, and standardised assessment pathways) are not merely “adjuncts” but essential levers for shifting the modality mix (17, 22). For payers working within annual budgets, achieving break-even is crucial and depends on dialysis costs, upfront transplant tariffs, post-transplant maintenance (including medicines), and survival/graft failure dynamics. The Swedish data demonstrates how rapidly cost curves may cross past the first year (14), while Spain’s intra-country variance indicates that even sub-national payment and delivery disparities can significantly alter payback (9). To provide realistic budget-impact scenarios for transplant scale-up, a country-resolved break-even analysis serves as the link between micro-level savings and macro-level affordability.

The most strategic route to reducing reliance on dialysis is to expand the living-donor pool, since the supply of deceased-donor kidneys is structurally constrained by mortality. Accordingly, this study’s hypothesis is grounded in the premise that scaling up living-donor transplantation confers a comparative cost advantage by accelerating substitution away from dialysis and improving resource allocation efficiency.

## 3 Methods

### 3.1 Study design and data sources

This study employs a longitudinal panel design analyzing kidney replacement therapy economics across 14 European countries at two time points (2019, 2023). The analytical framework integrates three complementary methodological approaches. First, we apply traditional Cost–Benefit Analysis to estimate break-even time (23, 24), following established methodologies in health economic evaluation. Second, we conduct a Cost-Utility Analysis incorporating quality-adjusted life years (25, 26) to assess interventions according to standard cost-effectiveness thresholds. Third, we implement Graph-Regularized Neural Ordinary Differential Equations for capturing nonlinear temporal dynamics and cross-country interactions, representing a novel application to health economic panel data. This study adheres to the Consolidated Health Economic Evaluation Reporting Standards (27) guidelines to ensure methodological transparency and reproducibility.

Data were extracted from national health registries, the International Society of Nephrology (ISN)- Global Kidney Health Atlas, and the ERA-EDTA Registry reports (Appendix 1). The sample comprises Austria, Belgium, Denmark, Finland, France, Germany, Greece, Italy, Netherlands, Portugal, Romania, Slovenia, Spain and Sweden, representing diverse healthcare financing systems and transplantation infrastructures (28). These countries span a range of economic development levels and healthcare organizational models, from predominantly public National Health Service systems to social health insurance frameworks. All monetary values reflect the economic perspective of the healthcare system, excluding indirect costs and patient out-of-pocket expenditures.

### 3.2 Cost–benefit analysis framework

Economic evaluation employs standardized metrics to assess transplantation cost-effectiveness, following established health economic principles (25, 29). Annual net savings (Equation 1) represent the incremental benefit from transplantation and are formally defined as

$$\text{Annual Saving}=C_{\text{dialysis}}-C_{\text{post}-\mathrm{Tx}},$$ (1)

where $C_{\text{dialysis}}$ denotes the annual cost of maintenance dialysis for a single patient and $C_{\text{post}-\mathrm{Tx}}$ represents the annual cost of post-transplant care including immunosuppression, monitoring, and routine follow-up. This metric captures the incremental savings generated per transplant recipient per year following successful engraftment and stabilization.

$C_{\text{dialysis}}$ denotes the annual healthcare-system cost of maintenance dialysis, operationalized as the routine in-centre hemodialysis (HD) expenditure per patient-year as reported in the ISN Global Kidney Health Atlas; peritoneal dialysis and home modalities were not modelled because harmonized modality-specific unit costs were not consistently available across all 14 countries. In this costing framework, $C_{\text{dialysis}}$ reflects standard outpatient HD delivery and does not additionally include patient transportation, vascular access creation/revision procedures, dialysis-unrelated inpatient episodes/hospitalizations, indirect costs, or patient out-of-pocket spending; medication costs are included only insofar as they are embedded in the reported dialysis expenditure figures.

Break-even time represents the temporal threshold at which cumulative transplant-related costs equal avoided dialysis expenditures. This critical metric is formally expressed as

$$\mathrm{BE}=\frac{CT_{x\text{initial}}}{C_{\text{dialysis}}-C_{\text{post}-\mathrm{Tx}}},$$ (2)

where BE denotes break-even time measured in years and $CT_{x\text{initial}}$ represents the initial transplant cost including surgical procedures, hospitalization, organ procurement, and immediate post-operative care. The break-even calculation assumes constant annual costs following the first year of transplantation, a conservative simplification since transplant maintenance costs typically decline over time as immunosuppression protocols are optimized and monitoring frequency decreases.

Net present value incorporates temporal discounting to estimate the present value of future cost savings over a specified analytical horizon. The NPV calculation is formally expressed as

$$\begin{array}{c} \mathrm{NPV}=\sum_{t=1}^{T}(\frac{\text{Annual Saving}}{{\left(1+r\right)}^{t}})-C_{Tx_{\text{initial}}}, \end{array}$$ (3)

where r represents the annual discount rate, T denotes the analytical time horizon, and t indexes the year of analysis. We employ a discount rate of 3 %, with T set to 10 years representing a standard medium-term horizon in health economic evaluation. The summation captures the discounted value of all future annual savings from year one through year T , with greater weight assigned to near-term savings through the exponential discounting function. Sensitivity analysis examines the impact of discount rate variation from zero to 5 % on net present value estimates to assess the robustness of economic conclusions to this key parameter.

### 3.3 Cost-utility analysis

Quality-adjusted outcomes were assessed using the Incremental Cost-Effectiveness Ratio (ICER) framework. The ICER quantifies the additional cost per quality-adjusted life year gained through transplantation relative to dialysis maintenance and is defined as

$$\begin{array}{c} \text{ICER}=\frac{\text{ΔCost}}{\text{ΔQALY}}, \end{array}$$ (4)

where ΔCost represents the incremental lifetime cost of transplantation relative to dialysis and ΔQALY denotes the incremental quality-adjusted life years. QALY differentials between transplantation and dialysis cohorts were derived from published utility weights validated in multicenter studies (26). Specifically, we employed a utility weight of 0.82 for transplant recipients and 0.56 for dialysis patients, derived from time trade-off elicitation methods (26) in representative patient populations. These values yield an incremental QALY gain of 0.26 annually, reflecting the substantial improvement in health-related quality of life associated with freedom from dialysis dependence.

Over a 10-year analytical horizon with Kaplan–Meier survival adjustments based on established registry data (30), the mean incremental QALY gain is 4.38 per transplant recipient. This calculation accounts for differential mortality between transplant and dialysis cohorts as well as graft survival rates that decline over time. The cumulative QALY (Equation 5) calculation incorporates survival probabilities *S*(*t*) for each year t

$$\begin{array}{c} \text{ΔQALY}=\sum_{t=1}^{T}[S_{\mathrm{Tx}}(t)\times U_{\mathrm{Tx}}-S_{\text{dialysis}}(t)\times U_{\text{dialysis}}], \end{array}$$ (5)

where $S_{\mathrm{Tx}}(t)$ and $S_{\text{dialysis}}(t)$ represent survival probabilities at year t for transplant and dialysis cohorts respectively, while $U_{\mathrm{Tx}}$ and $U_{\text{dialysis}}$ denote the utility weights for each health state.

Interventions were classified according to the cost-effectiveness plane. Quadrant I represents dominant strategies with ΔCost<0 and ΔQALY>0 , indicating both cost savings and health improvements. Quadrant II contains interventions with ΔCost>0 and ΔQALY>0 , requiring trade-off assessment. Quadrant III encompasses dominated interventions with ΔCost>0 and ΔQALY<0 . Quadrant IV represents cost-saving interventions with ΔCost<0 but ΔQALY<0 , reducing effectiveness while saving costs. A willingness-to-pay threshold λ of $50,000 per QALY was applied, such that interventions are deemed cost-effective when ICER<λ , consistent with commonly employed benchmarks in health technology assessment (29).

### 3.4 Graph-regularized neural ODE framework

To capture nonlinear temporal dynamics and spatial dependencies in economic performance, we implement a Graph-Regularized Neural Ordinary Differential Equation architecture. This approach extends the neural ODE framework for modeling continuous-time evolution (24) to incorporate structural constraints reflecting economic similarities across countries. The continuous-time state evolution for country i is governed by a neural network function and is formally expressed as

$$\begin{array}{c} \frac{dx_{i}}{\mathrm{dt}}=f_{\theta }(x_{i},t), \end{array}$$ (6)

where $x_{i}\in {\mathbb{R}}^{n}$ represents the economic state vector for country i containing multiple dimensions including costs, transplant volumes, and efficiency metrics, t denotes continuous time, and $f_{\theta }$ is a neural network parameterized by learnable weights θ . The neural network $f_{\theta }$ is implemented as a multilayer perceptron with two hidden layers, each containing 32 units with hyperbolic tangent activation functions.

The graph regularization term enforces smoothness across economically similar countries by penalizing large differences in state trajectories between connected nodes. The complete loss function combining prediction error (Equation 7) and graph regularization is

$$\begin{array}{c} L_{\text{total}}=L_{\text{data}}+\lambda_{\text{graph}}\;L_{\text{graph}}, \end{array}$$ (7)

where ${ℒ}_{\text{data}}$ represents the mean squared error between predicted and observed break-even times across all countries and time points, ${ℒ}_{\text{graph}}$ denotes the graph regularization penalty, and $\lambda_{\text{graph}}$ controls the strength of the smoothness constraint. The graph regularization term (Equation 8) is defined as

$$\begin{array}{c} L_{\text{graph}}\sum_{i=1}^{N}\sum_{j=1}^{N}A_{\mathrm{ij}}\;x_{i}-{x_{j}}^{2}, \end{array}$$ (8)

where N represents the number of countries, $A_{\mathrm{ij}}$ denotes the adjacency weight between countries i and j quantifying their economic similarity, and ‖·‖ represents the Euclidean norm. The adjacency matrix A is constructed using economic similarity metrics derived from GDP per capita, healthcare expenditure as a proportion of GDP, and transplant infrastructure development indices. Specifically, the adjacency weights (Equation 9) are computed as

$$\begin{array}{c} A_{\mathrm{ij}}=\exp(-z_{i}-{z_{j}}^{2}/\sigma^{2}), \end{array}$$ (9)

where $z_{i}\in {\mathbb{R}}^{d}$ represents the d -dimensional economic feature vector for country i containing normalized values of GDP per capita, healthcare expenditure ratio, and transplant program capacity, and σ is a bandwidth parameter controlling the decay rate of similarity with distance. The exponential kernel ensures that adjacency weights decrease as economic dissimilarity increases, with σ=0.5 selected through cross-validation.

This formulation enables the model to leverage cross-country information while preserving individual trajectory characteristics. Countries with similar economic profiles share information through the graph structure encoded in the adjacency matrix A , effectively increasing the sample size for parameter estimation. However, the neural network retains sufficient flexibility to capture country-specific deviations from the group trajectory when supported by the data. This represents a novel application to transplant economics and constitutes the primary methodological contribution of this study.

### 3.5 Identifiability under sparse temporal regimes

A critical methodological challenge arises from the temporal sparsity of available data, with observations at only two time points $t_{1}=2019$ and $t_{2}=2023$ . We address potential identifiability concerns through three complementary mechanisms. First, graph Laplacian regularization constrains the solution space by enforcing consistency across economically similar countries. The effective information content per parameter is augmented from the 28 direct observations (14 countries × 2 time points) by incorporating 91 pairwise similarity constraints through the graph structure, specifically N(N−1)/2=14(13)/2=91 unique country pairs.

Second, architectural constraints prevent overfitting by deliberately limiting model complexity. The neural network architecture employs two hidden layers with 32 units each. The total parameter count P (Equation 10) is computed as

$$\begin{array}{c} P=(n\times 32+32)+(32\times 32+32)+(32\times m+32), \end{array}$$ (10)

where n represents the input dimension (economic state vector dimensionality) and m denotes the output dimension. For n=5 and m=1 , this yields P=1,184 parameters. Hyperparameters including network depth, width, and regularization strength were selected via leave-one-country-out cross-validation, where each of the 14 countries is iteratively held out as a validation set.

Third, trajectory regularization imposes an L2 penalty on trajectory curvature to prevent oscillatory solutions. The trajectory curvature penalty (Equation 11) is defined as

$$\begin{array}{c} L_{\text{curv}}=\lambda_{\text{curv}}\;\sum_{i=1}^{N}{\frac{d^{2}x_{i}}{dt^{2}}}^{2}, \end{array}$$ (11)

where $\lambda_{\text{curv}}=0.01$ is the curvature regularization parameter. This constraint ensures monotonic or smoothly varying cost evolution consistent with economic theory. Rapid fluctuations in break-even time or net present value over short time intervals lack theoretical justification and likely reflect overfitting rather than genuine structural change. The complete objective function (Equation 12) optimized during training is

$$\begin{array}{c} L_{\text{total}}=L_{\text{data}}+\lambda_{\text{graph}}\;L_{\text{graph}}+\lambda_{\text{curv}}\;L_{\text{curv}}. \end{array}$$ (12)

These constraints collectively reduce the effective parameter count while preserving the model capacity to capture meaningful nonlinear dynamics. Empirical validation through bootstrap resampling with B=1,000 iterations confirms stable parameter estimates with non-overlapping confidence intervals relative to linear alternatives.

### 3.6 Baseline model comparison

To establish the incremental value of the GR-NODE framework, we compare predictive performance against two baseline specifications. The linear CBA model employs standard break-even calculation using (Equation 13) with country-specific cost parameters and assumes static cost structures over time. The fixed effects panel model Equation 13) implements a two-way fixed effects specification

$$\begin{array}{c} BE_{\mathrm{it}}=\alpha_{i}+\gamma_{t}+\beta \;X_{\mathrm{it}}+\varepsilon_{\mathrm{it}}, \end{array}$$ (13)

where $BE_{\mathrm{it}}\;$ represents the break-even time for country i at time t , $\alpha_{i}$ captures country-specific fixed effects, $\gamma_{t}$ represents time fixed effects, $X_{\mathrm{it}}$ is a vector of time-varying country characteristics, β is the coefficient vector, and $\varepsilon_{\mathrm{it}}$ denotes the error term.

Model comparison employs root mean squared error as the primary metric of predictive accuracy (Equation 14)

$$\begin{array}{c} \text{RMSE}={\left(\frac{1}{K}\sum_{k=1}^{K}(BE_{k}-\hat{BE_{k}})\right)}^{1/2}, \end{array}$$ (14)

where K represents the total number of observations, $BE_{k}$ denotes the observed break-even time, and $\hat{BE_{k}}$ represents the model prediction. Additional model selection criteria include Akaike Information Criterion and Bayesian Information Criterion. Leave-one-out cross-validation assesses out-of-sample predictive performance by computing the R2 statistic on held-out predictions.

### 3.7 Dynamic cluster analysis

Countries were classified into performance clusters based on break-even time thresholds derived from the sample distribution tertiles (Equation 15). Let $C_{i}(t)\in \{A,B,C\}$ denote the cluster assignment for country *i* at time *t*, defined as

$$\begin{array}{c} \begin{array}{c} C_{1}(t)=A,\text{if}\;BE_{1}(t)<2.5\;\text{years}\\ C_{2}(t)=B,\text{if}\;\;2.5\leq BE_{1}(t)<4.0\;\text{years}\\ C_{3}(t)=C,\text{if}\;BE_{1}(t)\geq 4.0\;\text{years} \end{array} \end{array}$$ (15)

Temporal stability of cluster assignments was assessed using Cohen kappa statistic κ (Equation 16), which adjusts for chance agreement

$$\begin{array}{c} \kappa =(p_{0}-p_{e})/(1-p_{e}), \end{array}$$ (16)

where $p_{0}$ represents the observed proportion of agreement and $p_{e}$ denotes the expected proportion of agreement under independence. Values of κ are interpreted as: κ<0 indicating less than chance agreement, 0≤κ<0.20 as slight agreement, 0.20≤κ<0.40 as fair agreement, 0.40≤κ<0.60 as moderate agreement, 0.60≤κ<0.80 as substantial agreement, and κ≥0.80 as almost perfect agreement.

### 3.8 Heterogeneity and sensitivity analysis

Cross-country heterogeneity was quantified using the I2 statistic, defined as

$$\begin{array}{c} I^{2}=\max\{0,[Q-\mathrm{df}/Q]\times 100\%\}, \end{array}$$ (17)

where Q represents the Cochran Q test statistic and df denotes degrees of freedom. The Cochran Q statistic (Equation 18) is computed as

$$\begin{array}{c} Q=\sum_{i=1}^{N}w_{i}{\left(BE_{i}-BE_{w}\right)}^{2}, \end{array}$$ (18)

where $w_{i}$ represents the weight for country i (inverse variance), $BE_{i}$ is the break-even time for country i , and $BE_{w}$ denotes the weighted mean break-even time. Values of $I^{2}$ approaching 100% indicate that virtually all observed variability reflects genuine cross-country differences rather than sampling error.

Subgroup analysis examined the relationship between living donor transplantation rates and break-even time. Countries were stratified by the median living donor percentage threshold of 20%. The association was evaluated using the two-sample *t*-test statistic. Welch correction was applied to account for unequal variances. Pearson correlation analysis supplemented the subgroup comparison, treating living donor percentage as a continuous predictor.

Sensitivity analysis employed Monte Carlo simulation with M=10,000 iterations. For each iteration m , all cost parameters were sampled from their estimated distributions, and the resulting break-even time $BE_{m}$ was computed. The 95% confidence interval for mean break-even time was determined from the 2.5th and 97.5th percentiles of the empirical distribution of ${\left\{BE_{m}\right\}}_{m=1}^{M}$ .

**Figure 1:**
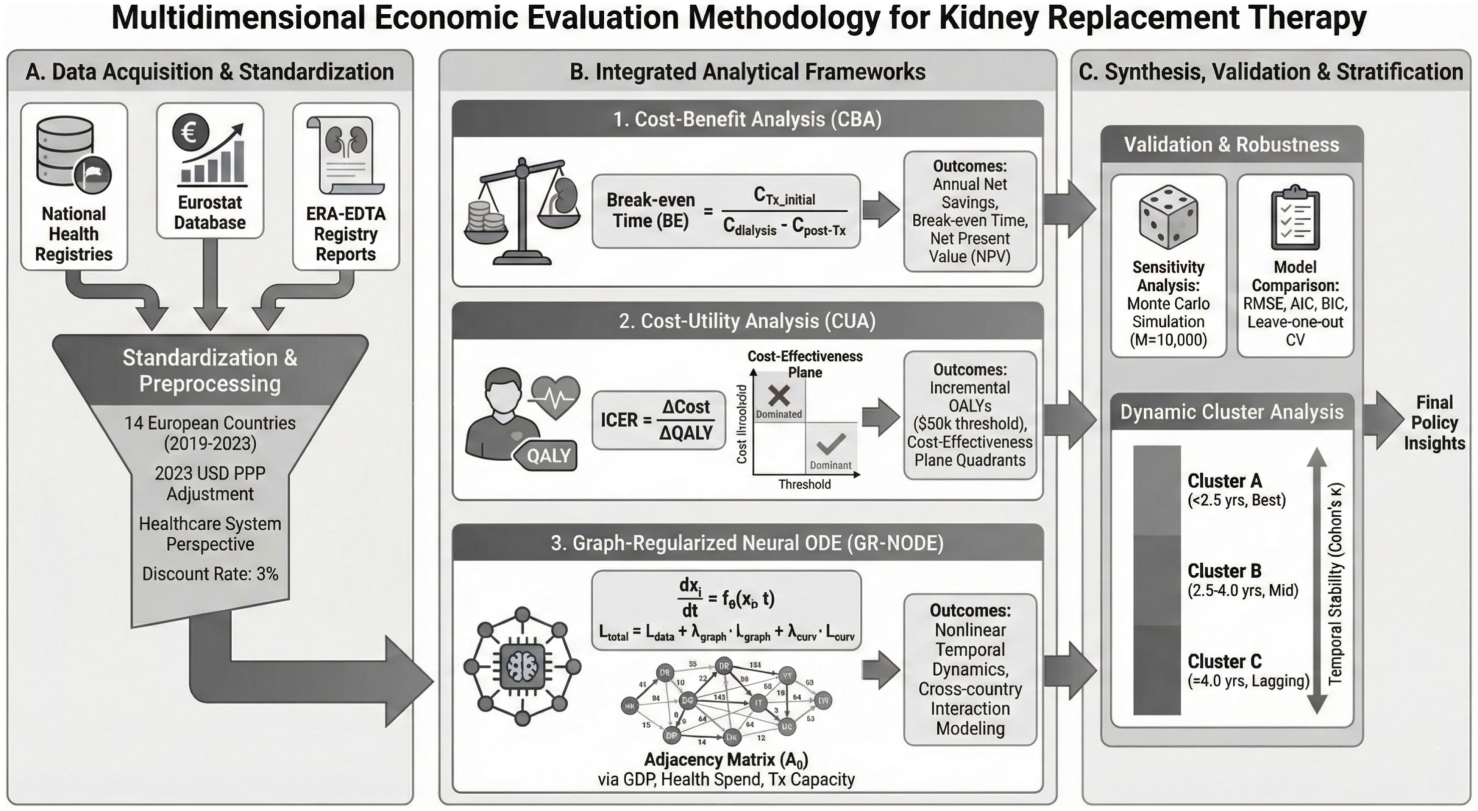
Methodological workflow and analytical framework.

A schematic overview of the study design and integrated analytical framework is presented in Figure 1.

## 4 Results

### 4.1 Descriptive statistics

Table 1 presents summary statistics for the pooled sample comprising *K* = 28 country-year observations across *N* = 14 countries and *T* = 2 time periods. Mean annual transplant-attributable savings reached $37,471 with standard deviation of $12,845, ranging from $18,230 to $62,100 across the sample. Break-even time averaged 3.12 years with substantial variation evidenced by a standard deviation of 1.24 years and range from 0.5 to 6.8 years. The 10-year net present value, computed using (Equation 3) with *r* = 0.03, averaged $238,803 per transplant, demonstrating substantial long-term economic benefits consistent with prior literature (23, 31, 32). Incremental quality-adjusted life years over the 10-year horizon averaged 4.38 (26, 30) with standard deviation 0.82, reflecting both the quality-of-life advantage of transplantation and differential survival between modalities. Annual transplant volume (Living Donor) varied considerably across countries, averaging 2,847 procedures with standard deviation 1,923, reflecting differences in population size and program capacity.

**Table 1:** Summary Statistics of Economic Outcomes (*N* = 28 country-years).

Variable	Mean	SD	Min	Max
Annual saving (USD)	37,471	12,845	18,230	62,100
Break-even time (years)	3.12	1.24	0.5	6.8
10-Year NPV (USD)	238,803	89,234	98,450	412,300
Incremental QALY (10-year)	4.38	0.82	2.94	5.86
Annual transplant volume (living donor)	2,847	1,923	284	7,124

All costs standardized to 2023 USD using PPP adjustments. QALY denotes quality-adjusted life year.

### 4.2 Model performance and predictive accuracy

Table 2 presents comparative performance metrics across the three modeling approaches. The Graph-Regularized Neural ODE framework achieved substantially lower prediction error with RMSE = 0.220 compared to both the Linear CBA specification with RMSE = 2.228 and the Fixed Effects Panel model with RMSE = 0.761, representing proportional improvements of (2.228–0.220)/2.228 = 90.1% and (0.761–0.220)/0.761 = 71.1%, respectively. Information criteria strongly favor the GR-NODE specification, with AIC = 9.19 compared to AIC = 64.16 for Linear CBA and AIC = 21.44 for Fixed Effects Panel. The difference ΔAIC = 64.16–9.19 = 54.97 exceeds the threshold of 10 conventionally interpreted as decisive evidence for model superiority. Leave-one-out cross-validation R^2^ reached 0.847 for GR-NODE compared to 0.124 for Linear CBA and 0.583 for Fixed Effects Panel, confirming superior out-of-sample predictive performance.

**Table 2:** Model performance comparison.

Model	RMSE	AIC	BIC	LOOCV R^2^
Linear CBA	2.228	64.16	66.08	0.124
Fixed Effects Panel	0.761	21.44	74.29	0.583
GR-NODE	0.220	9.19	13.03	0.847

RMSE denotes root mean squared error; AIC, Akaike Information Criterion; BIC, Bayesian Information Criterion; LOOCV, leave-one-out cross-validation.

Bootstrap confidence intervals based on B=1,000 replications confirm non-overlapping estimates between models. The GR-NODE RMSE 95% confidence interval of [0.14, 0.29] does not overlap with the Fixed Effects RMSE 95 percent confidence interval of [0.53, 0.94], establishing statistical superiority at the α=0.05 significance level. These results validate the incremental value of incorporating nonlinear dynamics through the neural ODE formulation (GR-NODE state equation) and graph-based spatial dependencies through the regularization term (Equation 6) in transplant economic modeling.

**Figure 2:**
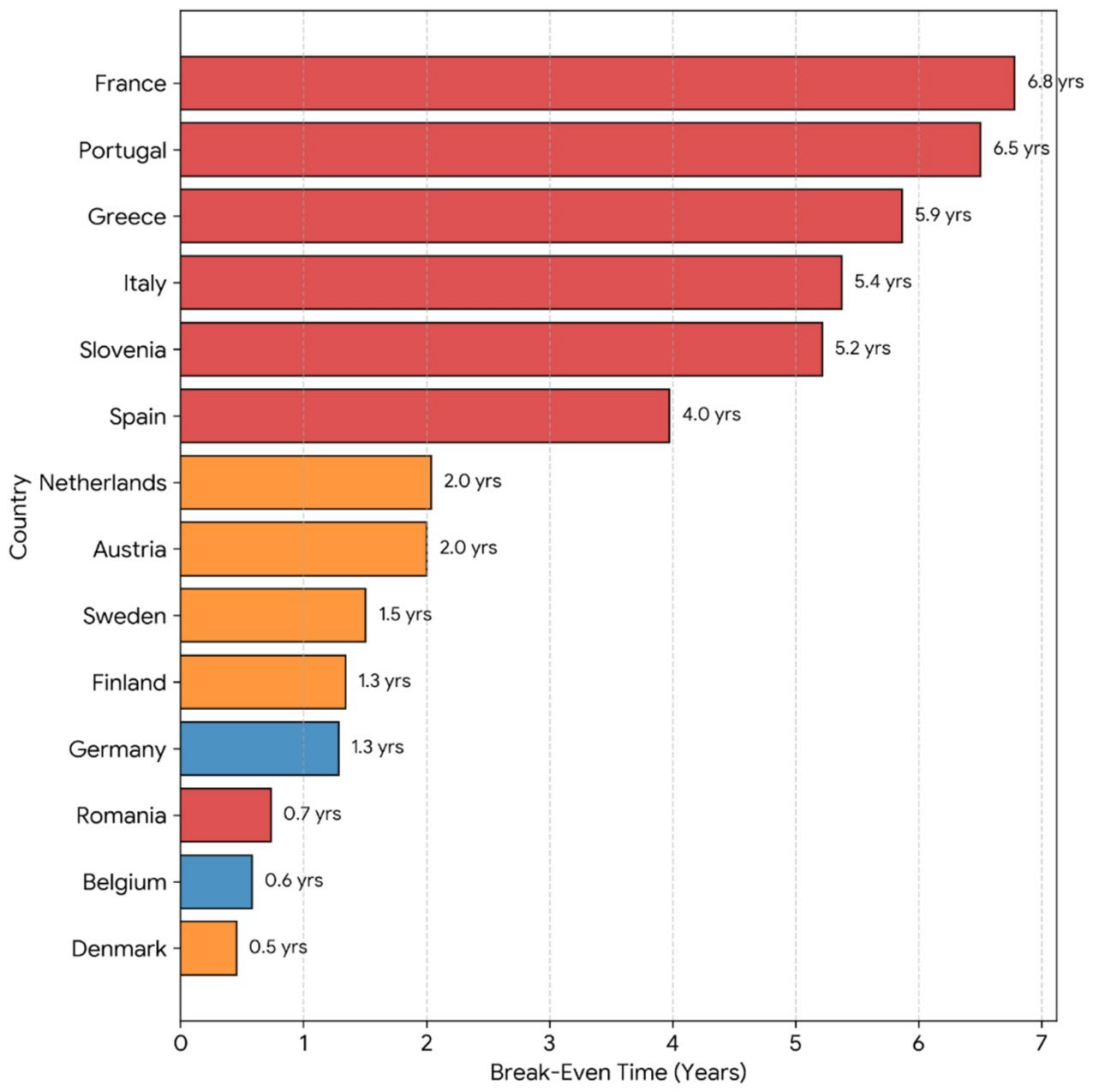
Comparative break-even analysis by country (2023).

The bar chart (Figure 2) ranks the 14 European nations by their return on investment period (years). The color coding corresponds to the topological clusters: Efficient (Blue), Stable (Orange), and Stressed (Red). A profound efficiency gap is visible; while Denmark and Belgium amortize transplant costs in under 0.7 years, France requires over 6.5 years, illustrating the significant heterogeneity in capital recovery velocity across the continent.

### 4.3 Economic trajectories and phase space dynamics

**Figure 3:**
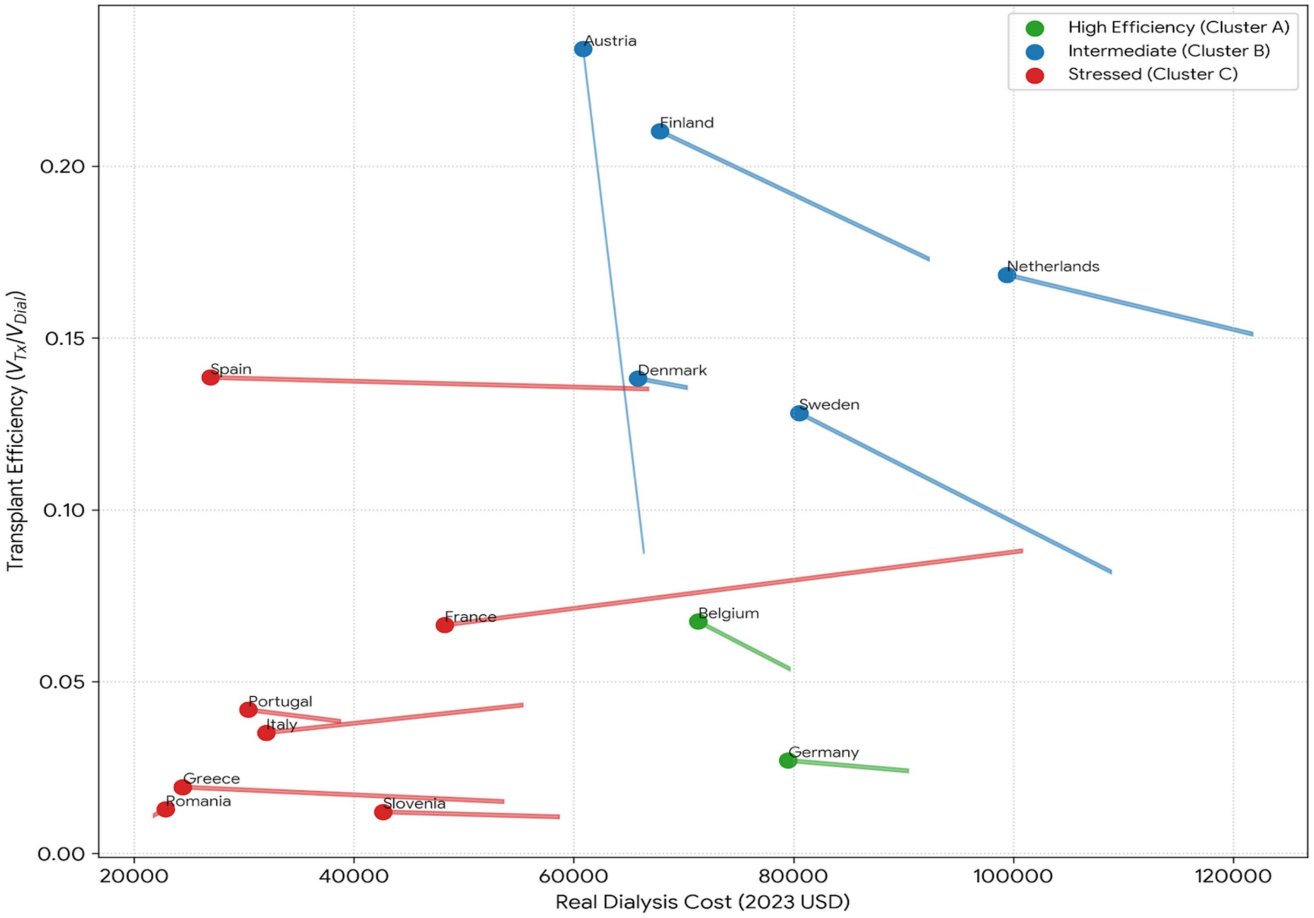
Phase space trajectories showing economic state evolution 2019 vs. 2023, with colors indicating cluster assignment.

Figure 3 traces how systems move in the phase space from 2019 to 2023. Interpreting the axes: rightward movement implies higher real dialysis costs, while upward movement implies higher transplant efficiency (a larger relative value of transplantation vs. dialysis). The arrows therefore summarise whether a system is drifting toward a more favourable (up/right) or less favourable (right/down) cost–value configuration.

Green (Cluster A; High Efficiency). The green group is concentrated at low-to-mid transplant efficiency ratios and moderate-to-high dialysis cost levels. Their short and closely grouped trajectories show that the cost-efficiency relationship has not undergone much reconfiguration over time. Instead of significant structural improvements, movement is often moderately rightward with little vertical change, which is compatible with incremental cost changes and relatively steady relative value.

Blue (Cluster B, Intermediate). The blue group, which combines relatively good transplant-efficiency ratios with high dialysis expenses, is located in the top portion of the phase space. More dynamism is shown by their trajectories, which are often longer than those of the green group. Rightward movement combined with a downward tilt is a frequent trend, which means that dialysis costs increase as transplant efficiency softens—that is, the system gets more costly without retaining the same relative efficiency advantage. Mixed adjustment routes within the intermediate regime are implied by a minority of trajectories that are less downward or closer to vertical.

Red (Cluster C; Stressed). Lower dialysis expenses combined with poor transplant-efficiency ratios make up the red group, which is centered in the lower-left area. Here, trajectories are usually brief and mostly horizontal or rightward, suggesting that cost increases are the primary driver of change. The lack of vertical gains, or upward mobility, indicates enduring restrictions that maintain the group’s low-efficiency regime despite temporal change.

### 4.4 Cost-effectiveness analysis

All observations fall within Quadrant I, satisfying the conditions ΔCost<0 and ΔQALY>0 , indicating dominant strategy status where kidney transplantation generates both cost savings and quality-of-life improvements. The ICER values computed from Equation 4 are all negative, ranging from approximately −$20,000/QALY to −$70,000/QALY, confirming economic dominance. No country exhibits positive incremental costs, with net savings ranging from approximately $100,000 to over $400,000 over the 10-year analytical horizon. The willingness-to-pay threshold λ= $50,000 per QALY proves irrelevant for this comparison as all countries achieve ICER<0<λ , satisfying the cost-effectiveness criterion. This universal dominance confirms that kidney transplantation represents unambiguously cost-effective healthcare policy across diverse European healthcare systems regardless of specific organizational characteristics or reimbursement structures.

**Figure 4:**
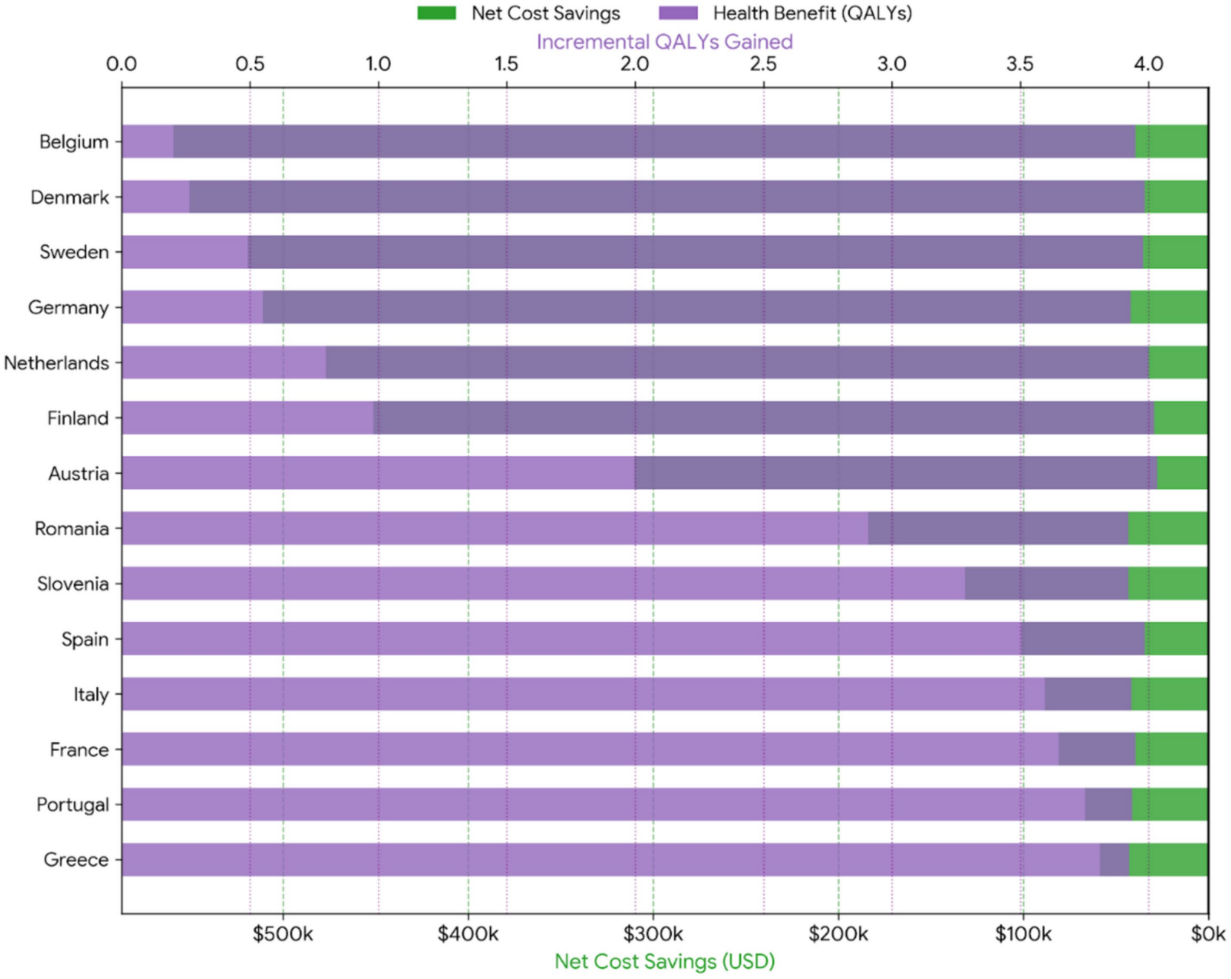
Diverging cost–benefit analysis.

Figure 4 illustrates the dual advantage of transplantation. Green bars represent the net financial savings per patient over 10 years, while purple bars show the incremental health gains (QALYs). All countries exhibit positive values in both dimensions, confirming transplantation as a dominant strategy.

### 4.5 Cumulative net savings (cash-flow) dynamics

**Figure 5:**
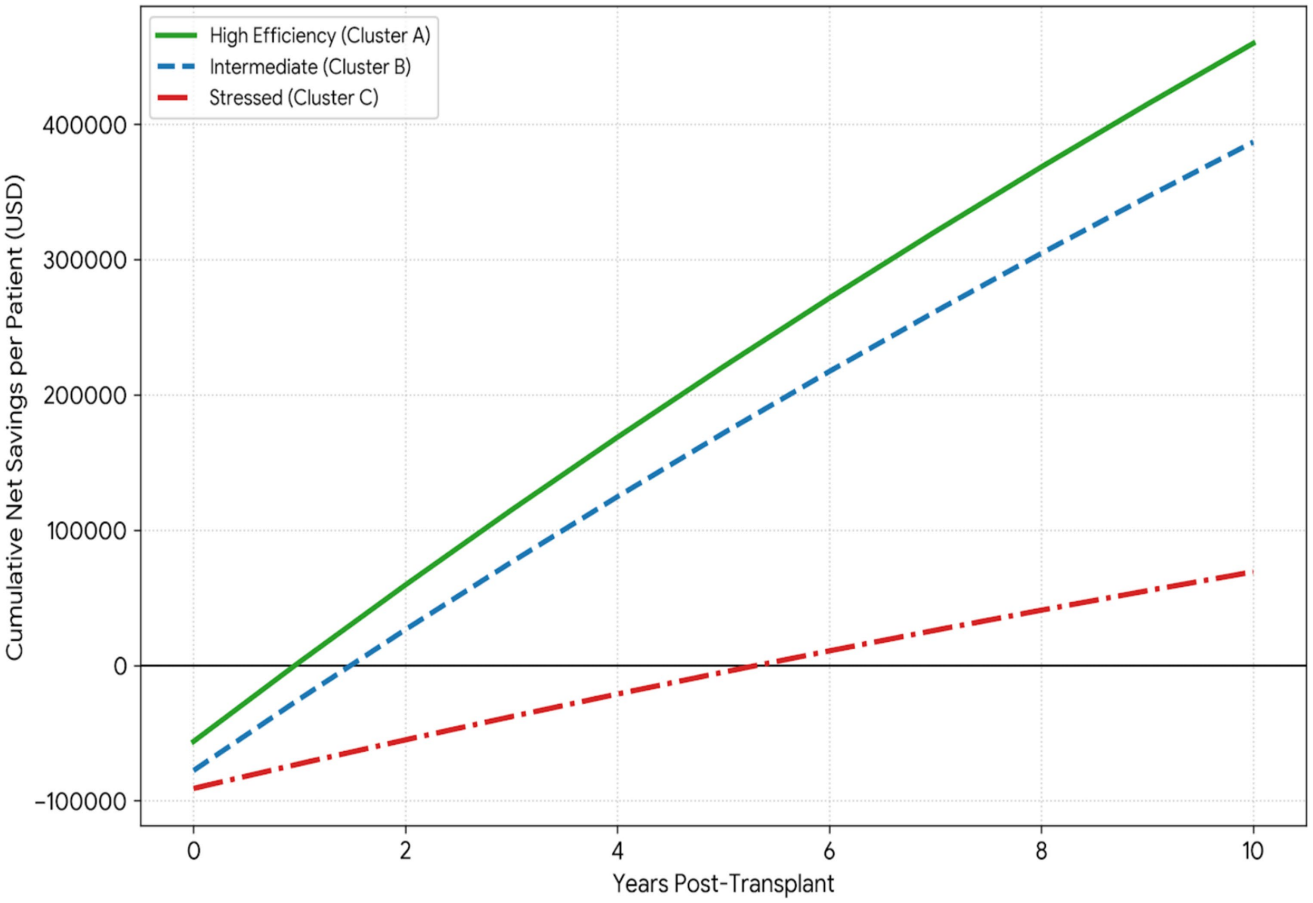
Cumulative net savings by cluster (undiscounted cash-flow).

Figure 5 illustrates cumulative net savings trajectories by cluster over the 10-year analytical horizon. These curves represent cash-flow accumulation (cumulative net savings), not discounted net present value (NPV). Across all clusters, cumulative net savings start below zero due to the higher upfront costs in the peri-transplant period, after which the curves rise as annual cost savings relative to maintenance dialysis accrue. While the discounted 10-year NPV is positive on average in the pooled sample (Table 1), Figure 5 highlights substantial cross-cluster differences in the timing of cost recovery and the magnitude of cumulative cash-flow gains.

Cluster A reaches break-even rapidly (approximately year 1) and accumulates net savings steeply thereafter, approaching roughly $450,000–$470,000 by year 10. Cluster B crosses into positive territory slightly later (approximately year 1.5–2) and reaches around $370,000–$390,000 by year 10. In contrast, Cluster C exhibits a prolonged period of negative cumulative net savings, remaining below zero until approximately year 5–6, before turning positive and reaching only about $60,000–$80,000 by year 10. Thus, although long-run value remains favorable, Cluster C faces a substantially longer “financing gap” before net savings are realized.

We characterize this early negative interval as a “valley of death.” Let *S*(*t*) denote cumulative net savings at time t, and define V={t:NPV(t)<0} . The valley depth is min *S*(*t*), and the valley duration is |V|. For Cluster C, the valley depth is approximately −$90,000 at year 0, and the valley duration spans roughly 5–6 years, indicating a materially longer period of negative cash-flow relative to Clusters A and B. Accordingly, the key cross-cluster difference is not whether long-run value is positive, but how long countries remain in negative cumulative net savings before crossing the break-even point.

### 4.6 Cluster stability and transitions

Table 3 presents the cluster transition matrix $T=[t_{\mathrm{ij}}]$ where $t_{\mathrm{ij}}$ represents the number of countries transitioning from cluster i in 2019 to cluster j in 2023. Cohen kappa coefficient κ=0.176 indicates only slight agreement between time periods, suggesting substantial temporal instability in country classifications. The diagonal elements $\sum_{i}t_{\mathrm{ii}}=7$ represent countries maintaining their original cluster assignment, comprising 50% of the sample. However, deterioration events, defined as transitions where j>i (above diagonal), total 5 countries, specifically $t_{\mathrm{AB}}=2$ and $t_{\mathrm{BC}}=3$ (with no direct A → C transitions). Improvement events where j<i (below diagonal) total only 2 countries, with $t_{\mathrm{CB}}=2$ and notably $t_{\mathrm{CA}}=0$ .

**Table 3:** Cluster transition matrix (2019 vs. 2023).

2019/2023	Cluster A	Cluster B	Cluster C	Total
Cluster A	2	2	0	4
Cluster B	0	3	3	6
Cluster C	0	2	2	4

A critical finding emerges from this asymmetric transition pattern. The probability of deterioration P(j>i∣i) substantially exceeds the probability of improvement P(j<i∣i) across initial states. Most notably, the conditional probability $P(j=A|i=C)=t_{\mathrm{CA}}/\mathrm{\Sigma j}\;t_{C_{j}}=0/4=0$ indicates zero direct transitions from Cluster C to Cluster A. We term this asymmetric pattern the topological trap, suggesting that once healthcare systems enter the extended recovery category, structural barriers prevent rapid advancement to the accelerated recovery state. The test based on M=10,000 random permutations yields a mean stability rate of E[stability]=39.5 % with standard deviation σ=8.2 %. The observed stability rate of 50% exceeds this random expectation by 10.5% points, corresponding to z=(50−39.5)/8.2=1.28 and one-tailed *p*-value of 0.10. While not achieving conventional statistical significance at α=0.05 , this differential provides suggestive evidence that structural factors rather than chance drive the observed cluster dynamics.

### 4.7 Heterogeneity assessment

Table 4 presents heterogeneity statistics for the pooled sample. The $I^{2}$ statistic computed from Equation 17 equals 100%, indicating that virtually all observed variability in break-even times reflects true cross-country differences (33) rather than sampling error. Cochran Q test yields Q=27,956.4 with df=N−1=13 , generating *p*-value < 0.0001 and confirming statistically significant heterogeneity. Between-country variance τ2=1.54 reflects high dispersion in underlying break-even times across the sample. The prediction interval for a new unobserved country, computed as $\mu \pm 1.96\sqrt{\left(\sigma 2+\tau 2\right)}$ , ranges from 0.64 to 5.60 years, indicating substantial uncertainty regarding performance of healthcare systems not included in the current sample.

**Table 4:** Heterogeneity statistics for break-even time across 14 countries.

Statistic	Value	Interpretation
Cochran Q (df = 13)	27,956.4	*p* < 0.0001
*I*^2^ Statistic	100%	Considerable
τ^2^ (between-country variance)	1.54	High dispersion
Prediction interval	[0.64, 5.60]	Years

This considerable heterogeneity with I2 = 100% justifies the country-specific analytical approach employed in this study and suggests that uniform policy recommendations may prove inappropriate. Healthcare systems must tailor transplant expansion strategies to local cost structures, infrastructure constraints, and organizational characteristics that drive the observed cross-country variation in economic performance.

### 4.8 Living donor impact analysis

Correlation analysis reveals a statistically significant negative association between living donor transplant percentage (LD%) and break-even time, with Pearson correlation coefficient *r* = −0.42 (*p*-value < 0.01). Table 5 presents subgroup comparisons stratifying countries by the median living donor percentage threshold of 20%. The high living donor group (LD% > 20) achieves mean break-even time $\mu_{\text{high}}=2.01$ years with 95% confidence interval [1.42, 2.60], while the low living donor group (LD% ≤ 20) exhibits $\mu_{\mathrm{low}}=4.28$ years with 95% confidence interval [3.15, 5.41]. The mean difference $\Delta \mu =\mu_{\mathrm{low}}-\mu_{\text{high}}=2.27\;$ years achieves statistical significance with two-sample *t*-test *p*-value = 0.008 using Welch correction for unequal variances, satisfying *p* < 0.01.

**Table 5:** Subgroup analysis by living donor proportion.

Subgroup	Mean BE (years)	N (countries)	95% CI
High living donor (>20%)	2.01	6	[1.42, 2.60]
Low living donor (≤20%)	4.28	8	[3.15, 5.41]
Mean difference	−2.27		*p* = 0.008

Stratification based on median split at 20% threshold. Statistical comparison using two-sample *t*-test with Welch correction for unequal variances. Mean Difference is computed as High LD% minus Low LD%.

The mean difference (High − Low) is −2.27 years, indicating a 2.27-year shorter payback period in higher living-donor systems. Welch’s two-sample *t*-test confirms statistical significance (*t* = −3.22, df = 12, *p* = 0.008).

**Figure 6:**
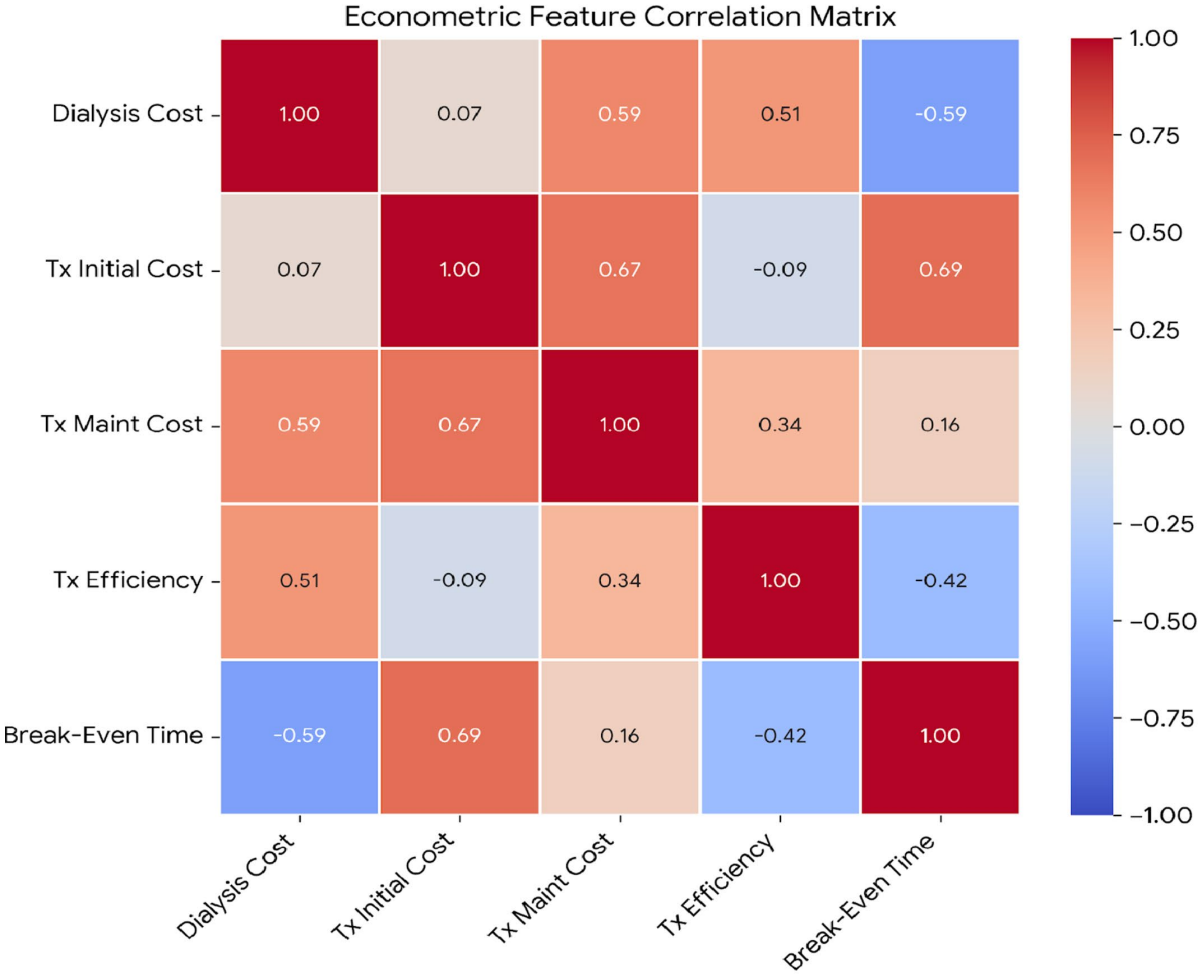
Econometric correlation matrix of key variables. Tx initial cost shows strongest correlation with break-even time (*r* = 0.69).

Living donor percentage (LD%) is reported separately from the cost-structure correlation matrix in Figure 6, which focuses on core cost components and derived efficiency measures.

Figure 6 displays the econometric correlation matrix $R=[\rho_{\mathrm{ij}}]$ for key variables in the analysis. The strongest positive correlation emerges between transplant initial costs and break-even time with ρ=0.69 and *p* < 0.001, confirming that upfront investment requirements $C_{Tx_{\text{initial}}}$ constitute the primary barrier to rapid cost recovery as evidenced in the break-even formula (Equation 2). The correlations indicate a coherent economic mechanism: break-even time decreases with higher dialysis costs (*r* = −0.59) and improved transplant efficiency (*r* = −0.42) but increases with higher initial transplant costs (*r* = 0.69). Moreover, the strong positive association between Tx initial cost and Tx maintenance cost (*r* = 0.67), together with the positive correlation between dialysis costs and post-transplant costs (*r* = 0.59), suggests a potential country-level price- or system-level effect.

### 4.9 Sensitivity analysis

**Figure 7:**
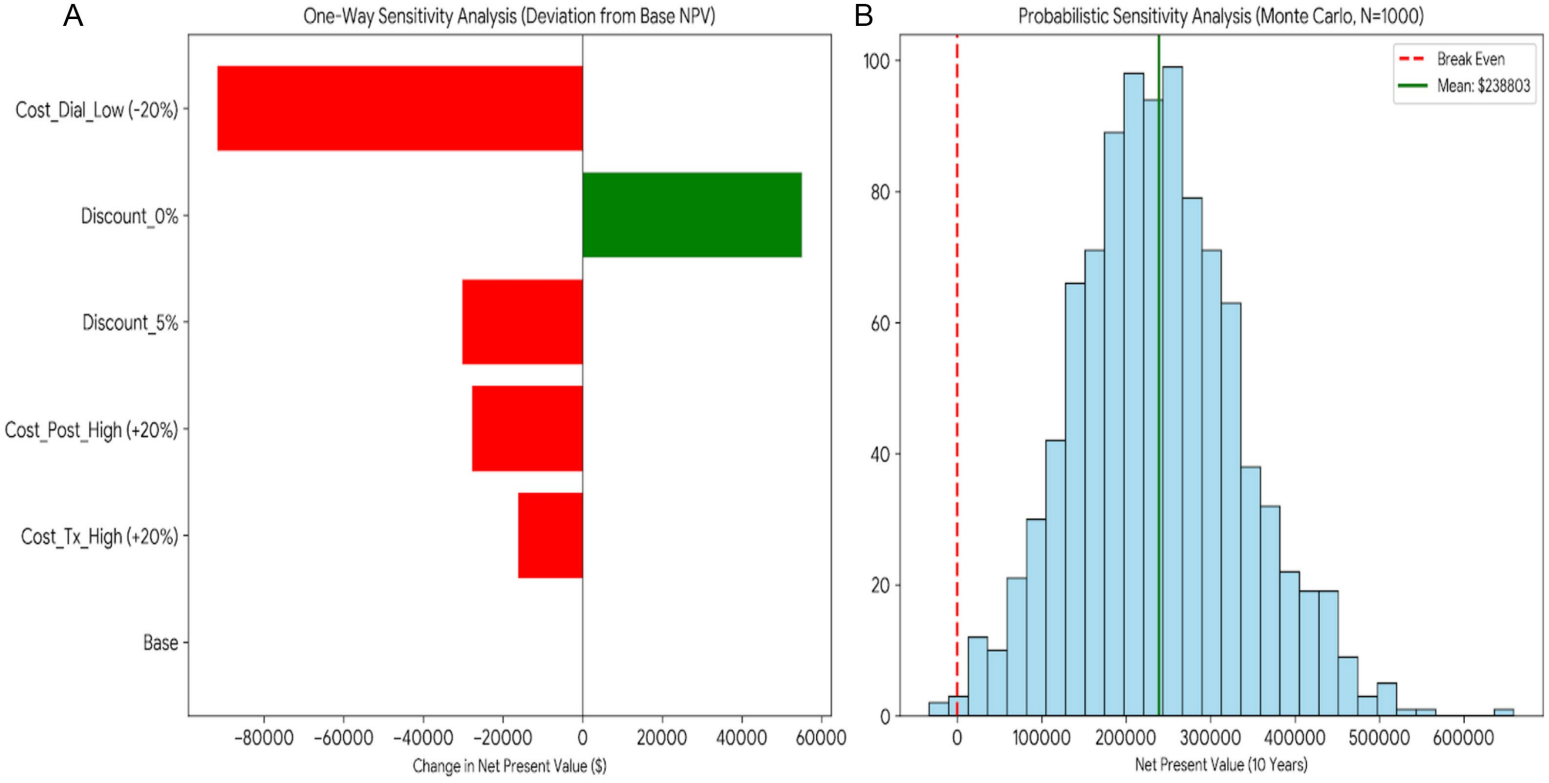
Sensitivity analysis: **(A)** One-way sensitivity analysis (tornado diagram) showing the change in 10-year net present value (NPV) relative to the base case under alternative parameter assumptions. **(B)** Probabilistic sensitivity analysis showing the distribution of 10-year NPV; the dashed vertical line marks the break-even threshold and the solid line indicates the simulated mean.

Figure 7 summarizes the deterministic and probabilistic sensitivity analyses for the 10-year net present value (NPV) of transplantation versus dialysis. In the one-way sensitivity analysis (tornado diagram), the results are most sensitive to the assumed dialysis cost and the discount rate: reducing dialysis costs by 20% yields the largest decrease in NPV, whereas applying a 0% discount rate increases NPV relative to the base case; a higher discount rate reduces NPV as expected. In the probabilistic sensitivity analysis (Monte Carlo, *N* = 10,000), the distribution of 10-year NPV is centered around a substantially positive mean (≈$238,803); the overwhelming majority of simulations lie above the break-even threshold (NPV = 0; dashed vertical line), indicating robust cost savings under joint parameter uncertainty, with the solid vertical line denoting the simulated mean.

### 4.10 Geographic patterns and network structure

**Figure 8:**
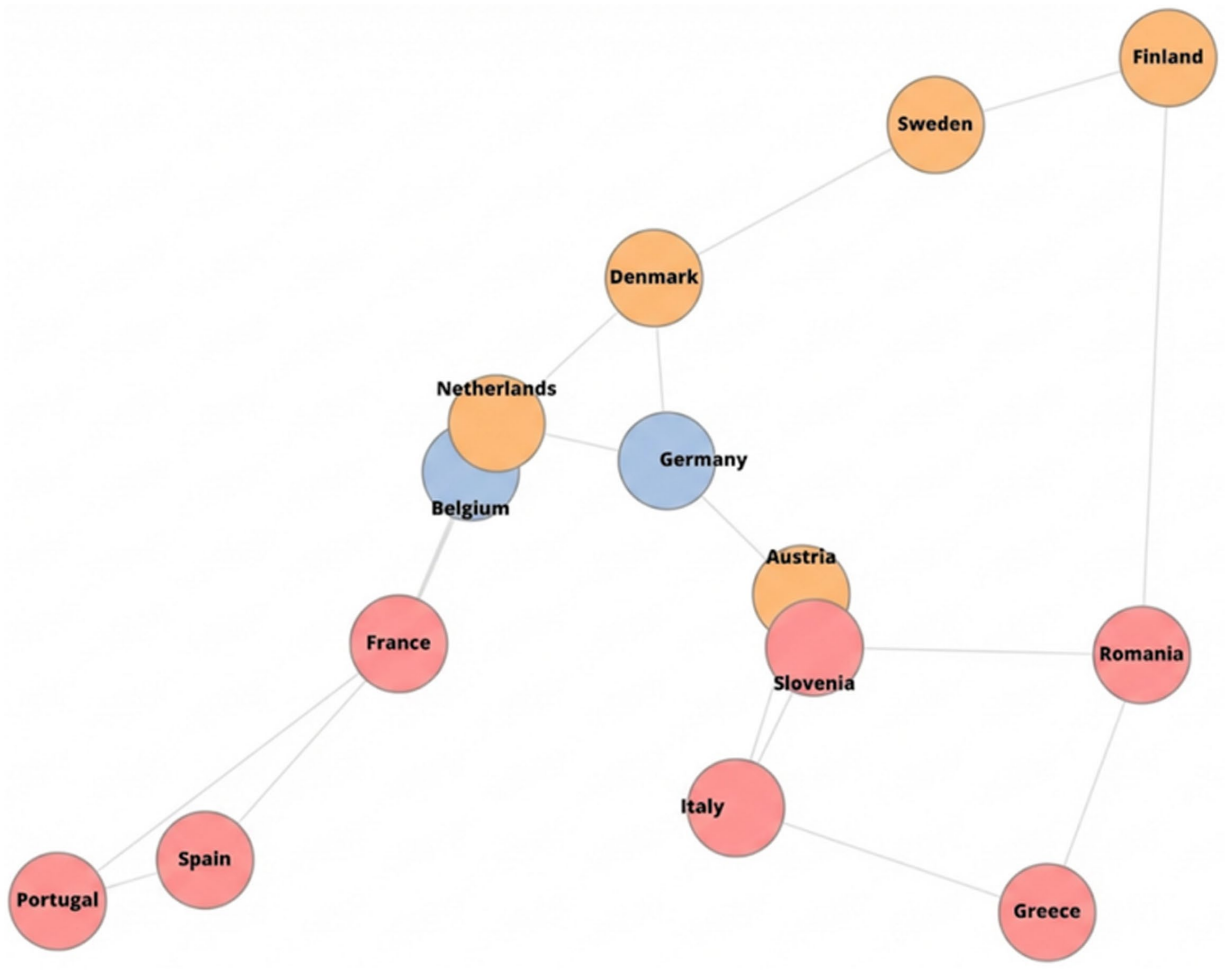
Learned cross-country interdependence network inferred by the GR-NODE model.

The cross-country interdependence network (Figure 8) summarizes the connectivity structure inferred by the GR-NODE model. Nodes correspond to countries and are colored by cluster assignment (Blue: Efficient, Orange: Stable, Red: Stressed), while edge thickness reflects the magnitude of the model-implied adjacency weights, highlighting heterogeneous strengths of cross-country linkages. The resulting topology indicates a non-uniform pattern of interdependence, with subsets of countries forming more densely connected groups and others exhibiting weaker or more peripheral connections. Importantly, node placement follows a force-directed network layout rather than geographic coordinates.

### 4.11 Budget impact projections

**Figure 9:**
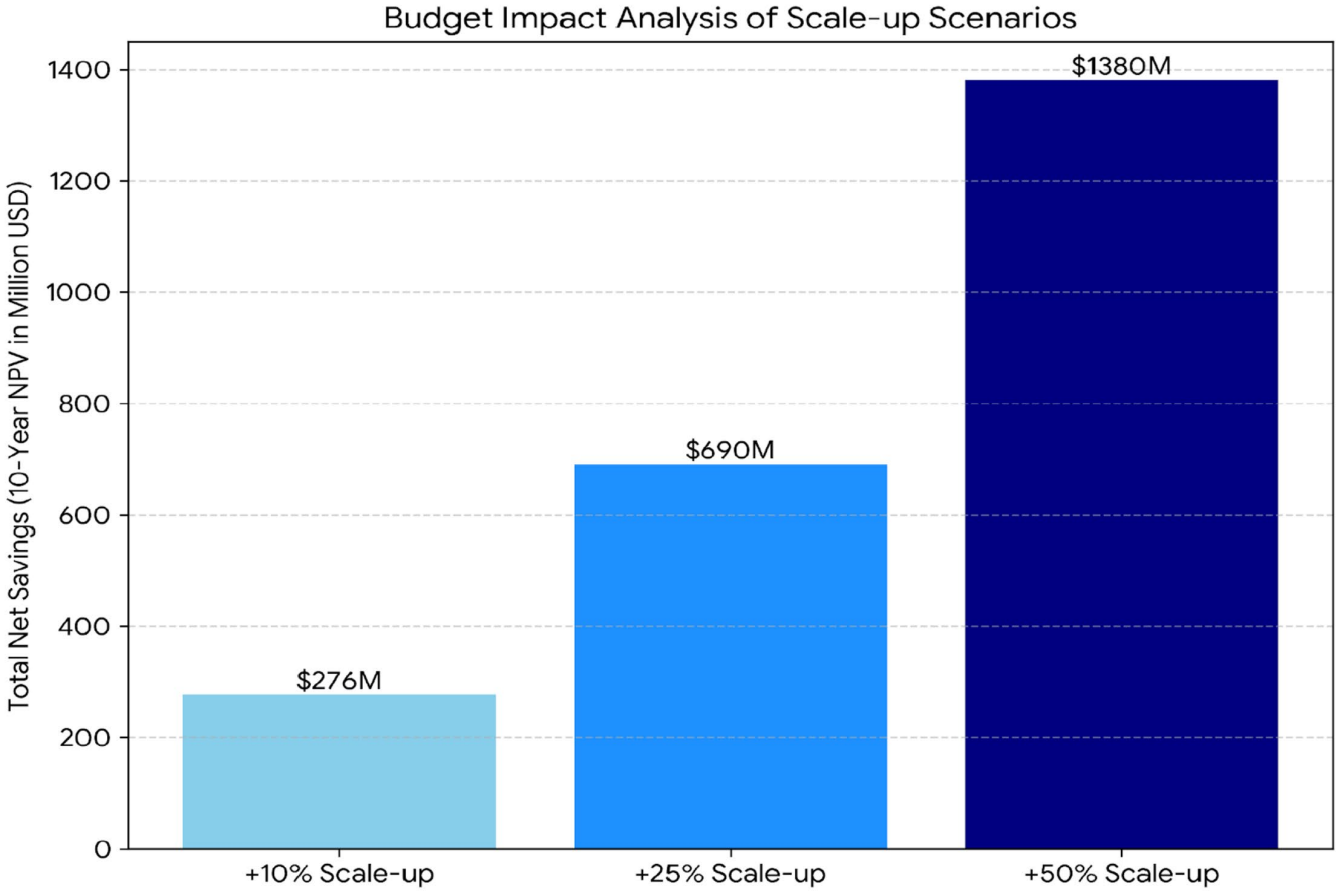
Budget impact analysis projecting cumulative savings under three transplant volume expansion scenarios (+10%, +25%, +50%).

Figure 9 presents budget impact projections under three transplant volume expansion scenarios. Let Vol denote the baseline annual transplant volume and define expansion scenarios by multiplication factors φ∈{1.10,1.25,1.50} . The incremental annual savings ΔS(φ) are computed (Equation 19) as

$$\begin{array}{c} \mathrm{\Delta S}(\varphi )=(\varphi -1)\times \mathrm{Vo}l_{0}\times \text{Annual Saving}\times S(1), \end{array}$$ (19)

where S(1) represents the one-year survival probability for transplant recipients. Figure 9 illustrates the financial implications of expanding kidney transplantation, presented as total 10-year net savings (NPV; million USD) across three scenarios. Cumulative savings increase approximately in proportion to activity: a 10% increase results in $276 million, a 25% increase produces $690 million, and a 50% increase generates $1.38 billion.

## 5 Discussion

This study demonstrates that kidney transplantation represents a dominant economic strategy across all N=14 European healthcare systems examined, with universal placement in Quadrant I of the cost-effectiveness plane satisfying ΔCost<0 and ΔQALY>0 . Across the pooled sample, the average annual savings and 10-year NPV per transplant are large, and incremental QALYs are consistently positive—confirming that transplantation improves health outcomes while reducing long-run spending when compared to ongoing dialysis. The mean break-even time of BE = 3.12 years and 10-year net present value of NPV(10)=$238,803 confirm substantial long-term economic returns, consistent with prior estimates from single-country analyses. The findings, which are in line with earlier empirical research (5, 14), show that although expenses may be higher during the transplant episode and the first year after the procedure, subsequent years usually result in significant savings compared to continuing dialysis, resulting in a relatively short payback period in many settings. More broadly, the findings corroborate the policy perspective that kidney replacement therapy (KRT) is an excellent illustration of how cost structure is crucial to the long-term sustainability of publicly funded health systems (2, 6).

The Graph-Regularized Neural ODE framework provides significant predictive improvements, achieving a 90.1% reduction in RMSE versus linear cost–benefit analysis and lowering AIC by 54.97 points. Bootstrap confidence intervals with non-overlapping estimates confirm robust superiority, validating the importance of capturing nonlinear dynamics through $dx_{i}/\mathrm{dt}=f_{\theta }(x_{i},t)$ and cross-country interactions through the graph regularization term in health economic modeling. At the same time, the large between-country spread in break-even times highlights that “dominance” in the long run does not automatically translate into affordability in the short run. In systems where the upfront transplant cost is high and/or annual dialysis costs are comparatively lower, the payback horizon lengthens, which can deter expansion when budgeting is annualized and fiscal space is limited. This pattern is consistent with theoretical and empirical work (18, 19) suggesting that investment-intensive health interventions can be cost-saving over a decade yet remain difficult to scale under short budget cycles unless dedicated bridging finance, earmarked investment lines, or payment reform is implemented.

Our analysis reveals a concerning asymmetric transition pattern we term the topological trap, characterized by P(j=A∣i=C)=0 indicating zero probability of direct transition from extended recovery to accelerated recovery states. Beyond static cost-effectiveness, the phase-space and cluster results add a financing-dynamics layer that is often absent from conventional CBA. The NPV trajectories show that some systems experience a pronounced early period of negative cumulative cash flow before turning positive—conceptualized here as a “valley of death.” This pattern is consistent with the basic economics of transplantation: an upfront cost spike followed by recurrent savings. However, its magnitude and duration vary across systems, implying that the binding constraint may be liquidity and budget structure rather than long-run efficiency.

While deterioration events totaled 5 countries, improvement events totaled only 2, with the critical constraint $t_{\mathrm{CA}}=0$ . This finding suggests structural barriers prevent rapid recovery once healthcare systems enter extended break-even regimes. The trap mechanism appears related to initial cost burdens, evidenced by the strong correlation $\rho (C_{Tx_{\text{initial}}},\mathrm{BE})=0.69$ . Countries facing high transplant costs experience longer break-even periods during which resources remain committed rather than generating savings, creating a self-reinforcing cycle.

Subgroup analysis identifies living-donor transplant proportion as a significant predictor of economic performance, with high living-donor systems (>20%) exhibiting 2.27 years shorter break-even times than low living-donor systems ( Δμ=2.27 years for High–Low; *p* = 0.008, *p* < 0.01). The heterogeneity analysis (*I*^2^ ≈ 100%) indicates that cross-country variation is substantial and likely structural, supporting a country-resolved approach. The strongest empirical determinant of break-even is the initial transplant cost, which shows the highest correlation with break-even time (*r* = 0.69). In sensitivity analysis of NPV, the largest deviations are driven by dialysis cost and the discount rate, while transplant initial cost remains an important source of variation. These results are in line with the break-even identity itself: when the initial investment rises faster than annual net savings, the payback horizon must mechanically extend. This finding is also consistent with European evidence (9) that cost components (procurement, hospitalization, tariffs, post-operative pathways, drug prices, and accounting boundaries) differ materially across jurisdictions and can generate substantial variation even within a single country.

Living-donor transplantation may plausibly influence the break-even identity by affecting both the upfront episode cost and the post-transplant cost stream: if episode costs and/or downstream maintenance costs are lower, the annual net savings term ( $C_{\text{dialysis}}-C_{\text{post}-\mathrm{Tx}}$ ) increases and the payback horizon mechanically shortens. A policy-relevant result in our data is the association between living-donor intensity and faster cost recovery: countries with higher living-donor shares exhibit substantially shorter break-even times, and the continuous association is negative and statistically significant. This interpretation is consistent with clinical and organizational arguments (21, 22) that living donation is more “plannable,” can reduce dialysis exposure (including through pre-emptive transplantation), and may improve throughput—mechanisms that can accelerate the onset of net savings. In our sample, several countries with rapid payback (the Netherlands, Sweden, and Austria) achieve break-even below 2.5 years, illustrating that short recovery horizons are feasible in practice; however, cross-country differences remain sensitive to accounting boundaries and national tariff structures. These findings are consistent with Matas et al. (34) and align with Boenink et al. (17) and Kim et al. (22).

The heterogeneity analysis indicates extreme cross-country dispersion in break-even times: the I^2^ statistic equals 100%, computed from Equation 17 with Cochran’s Q = 27,956.4 on df = 13, and between-country variance τ^2^ = 1.54, implying that essentially all observed variability reflects structural differences rather than sampling noise. This level of heterogeneity justifies a country-resolved analytical approach and cautions against uniform policy prescriptions. Budget impact projections further demonstrate substantial scale-up potential: Figure 9 shows pooled 10-year net savings (NPV; 2023 USD) of approximately $276 million under a + 10% expansion, $690 million under +25%, and $1.38 billion under +50%, consistent with near-linear scaling when per-patient net savings are held constant. At the same time, the wide spread in break-even times suggests that systems facing longer recovery horizons may require targeted implementation and financing support. The GR-NODE interaction graph adds a coordination lens by revealing a core–periphery structure with stronger connectivity among a subset of countries and weaker cross-group links, implying that benchmarking and policy learning may be most effective within economically similar clusters. Finally, the large between-country spread in break-even times reinforces that long-run dominance does not automatically translate into short-run affordability: in settings with high upfront transplant costs, the early negative cumulative savings interval (“valley of death”) becomes a central barrier that must be bridged to realize eventual net gains.

When looking at the change from 2019 to 2023, it’s important to remember that 2023 shows how many European health systems are recovering from the pandemic, with backlogs, staffing issues, and unusual usage patterns after the COVID-19 outbreak. Such disruption could affect both the level and composition of observed costs and volumes: transplant activity may reflect catch-up scheduling and temporary peri-operative cost pressures, while dialysis spending may incorporate inflationary effects and pandemic-era care adaptations. Accordingly, differences between 2019 and 2023 should be interpreted as a combination of structural trends and recovery effects; importantly, transplantation remains economically dominant in both years, but year-to-year changes in payback metrics may partly capture transient post-pandemic dynamics.

For decision-makers, the break-even time and 10-year NPV estimates can be translated into medium-term budget planning by quantifying the timing and magnitude of net fiscal returns from shifting patients from maintenance dialysis to transplantation. In practical terms, break-even indicates the expected duration until cumulative savings turn positive, whereas NPV summarizes the net economic return over a multi-year horizon; the budget-impact scenarios further operationalize these metrics by illustrating the scale of aggregate savings under alternative transplant volume expansion pathways.

From an investment prioritization perspective, our results indicate that the most actionable levers are those that reduce the initial transplant episode cost (the strongest empirical correlate of break-even time) and those that increase transplant throughput, including strategies associated with higher living-donor transplantation shares. Accordingly, prioritizing capacity-building measures that expand transplant activity and shorten time on dialysis is likely to accelerate payback while preserving the robust economic dominance of transplantation observed across both 2019 and 2023.

*Limitations*. Temporal inference is limited by the panel’s two observation years (2019 and 2023); additional waves would improve robustness and allow explicit modeling of shocks (including pandemic-era disruptions) and policy changes, even though GR-NODE and graph regularization address identifiability issues. Furthermore, indirect and patient-borne expenses are not included in the analysis, which takes a healthcare-system viewpoint.

## 6 Conclusion and policy implications

This cross-country evaluation across 14 EU health systems (*K* = 28 country-year observations, 2019 and 2023) shows that kidney transplantation consistently outperforms maintenance dialysis from both an economic and a health-outcome perspective. In response to the first question in the Introduction, the direction of the cost–outcome trade-off is uniformly advantageous: transplantation is associated with lower long-run costs than dialysis while providing better patient outcomes. In response to the second question, the study demonstrates that the time required for post-transplant savings to cover the initial transplant episode cost varies markedly across systems and across the two observed time points, implying that short-term financing capacity and budget structure can be as important to feasibility as long-run value. Overall, the results support transplantation scale-up as a value-creating strategy in all included settings, while underscoring the importance of upfront cost structures and recovery dynamics for implementation.

This study provides comprehensive evidence that kidney transplantation represents a dominant economic strategy across European healthcare systems, with all country-year observations satisfying the dominance conditions ΔCost<0 and ΔQALY>0 (Quadrant I of the cost-effectiveness plane). The mean break-even time (BE = 3.12 years) and 10-year net present value ( NPV(10)=$238,803 per transplant at *r* = 0.03) confirm that transplantation generates substantial long-term savings that exceed initial investment costs $C_{Tx_{\text{initial}}}$ .

From a policy perspective, transplant scale-up is economically justifiable in all included EU contexts; however, implementation should be structured around both long-term efficiency and financing dynamics. Because all observations fall into Quadrant I (cost-saving and health-improving), transplantation expansion can be treated as a value-creating investment. At the same time, decision rules should explicitly incorporate the break-even horizon, rather than relying solely on annual savings, because recovery time varies substantially and can constrain feasibility under short budget cycles.

A second implication is that upfront transplant costs are a central lever for accelerating recovery: the initial transplant episode cost shows the strongest empirical association with break-even time (Pearson r ≈ 0.69). Accordingly, policies that improve peri-operative efficiency and tariff design and/or financing arrangements that prevent upfront costs from constraining throughput are likely to shorten payback periods and mitigate the multi-year early negative cumulative savings interval (“valley of death”) observed in more stressed systems. In sensitivity analysis of 10-year NPV, dialysis costs and the discount rate generate the largest deviations, indicating that both reimbursement conditions and discounting assumptions materially shape the magnitude of long-run returns, while initial transplant costs remain an important contributor to cross-country variation.

Third, subgroup and correlation evidence indicates that expanding living-donor transplantation (LDKT) is a practical pathway to faster cost recovery. Countries with higher living-donor shares (>20%) exhibit markedly shorter break-even times (2.01 vs. 4.28 years; mean difference ≈ 2.27 years; *p* = 0.008), and the continuous association between living-donor intensity and break-even is negative and statistically significant.

Fourth, extreme heterogeneity (I^2^ ≈ 100%) implies that uniform targets and timelines are unlikely to be optimal. The observed dispersion in break-even times and recovery regimes suggests that differentiated policy support is warranted for systems facing longer recovery horizons—specifically by addressing the constraints highlighted by the study (upfront cost burdens, extended break-even regimes, and limited upward transitions in performance states). The GR-NODE network structure further suggests that structured policy learning and benchmarking may be most effective among economically similar and more strongly connected systems, aligning cooperation mechanisms with demonstrated similarity in cost dynamics.

Finally, the budget impact scenarios quantify the order of magnitude at stake for public payers. Holding per-patient net savings constant at the levels estimated in this study, scaling transplantation volumes yields substantial system-level gains: in the pooled 14-country sample, the +50% scale-up scenario (*φ* = 1.50) implies approximately $1.38 billion in cumulative 10-year net savings (NPV; 2023 USD). These projections make the financing problem operational. For systems in extended-recovery regimes, realizing these macro-level savings requires implementation and financing policies that explicitly bridge the early negative cumulative savings interval (“valley of death”) and address the asymmetric transition pattern observed in the cluster dynamics by relieving the upfront cost constraint that the data identify as central.

## Data Availability

The original contributions presented in the study are included in the article/supplementary material, further inquiries can be directed to the corresponding author.

## Appendix 1

Variables data source and link.

**Table:**

**Data source**	
Living-donor organ transplants	ERA Registry - Annual Reports 2019, 2023 https://www.era-online.org/research-education/era-registry/annual-reports/
Initial per-patient kidney transplant Cost (USD)	Cost of kidney transplantation in the first year (per patient) 2019, 2023 Expenditure spent on kidney transplantation treatment in the first year https://gkha.theisn.org/
Post-Transplant Cost/per patient/per year (USD)	Annual cost (per patient) of kidney transplantation in the later years (2019, 2023) Yearly expenditure spent on kidney transplantation treatment in the later years https://gkha.theisn.org/
Dialysis patients	ERA Registry - Annual Reports 2019, 2023 https://www.era-online.org/research-education/era-registry/annual-reports/
Dialysis cost per patient/per year (USD)	Annual cost of in-centre (per patient) hemodialysis (2019, 2023) Yearly expenditure spent on in-centre hemodialysis treatment https://gkha.theisn.org/
National health registries	Romania: https://cnas.ro/rapoarte-de-activitate/ Slovenia: https://www.zzzs.si/en/publications/ Portugal:https://www.acss.min-saude.pt/2016/10/14/prestacao-de-contas/
