# Supplementary material for: Kidney transplantation versus maintenance dialysis in 14 EU countries: cost savings, payback time, and budget impact
Source: Front Public Health. 2026 Mar 10;14:1801439. doi: 10.3389/fpubh.2026.1801439 (PMC13008983; doi:10.3389/fpubh.2026.1801439)
Supplement: Supplementary file 1 [file Table_1.docx]

**Appendix 1. Variables data source and link**

| **Data source** | |
| --- | --- |
| Living-donor organ transplants | ERA Registry - Annual Reports 2019, 2023  <https://www.era-online.org/research-education/era-registry/annual-reports/> |
| Initial per-patient kidney transplant Cost (USD) | Cost of kidney transplantation in the first year ( per patient) 2019, 2023  Expenditure spent on kidney transplantation treatment in the first year  <https://gkha.theisn.org/> |
| Post-Transplant Cost / per patient / per year (USD) | Annual cost ( per patient ) of kidney transplantation in the later years (2019, 2023)  Yearly expenditure spent on kidney transplantation treatment in the later years  <https://gkha.theisn.org/> |
| Dialysis patients | ERA Registry - Annual Reports 2019, 2023  <https://www.era-online.org/research-education/era-registry/annual-reports/> |
| Dialysis cost per patient / per year (USD) | Annual cost of in-centre (per patient ) hemodialysis (2019, 2023)  Yearly expenditure spent on in-centre hemodialysis treatment  <https://gkha.theisn.org/> |
| National health registries | Romania: <https://cnas.ro/rapoarte-de-activitate/>  Slovenia: <https://www.zzzs.si/en/publications/>  Portugal:<https://www.acss.min-saude.pt/2016/10/14/prestacao-de-contas/> |
